# The GPCR–β-arrestin complex allosterically activates C-Raf by binding its amino terminus

**DOI:** 10.1016/j.jbc.2021.101369

**Published:** 2021-10-30

**Authors:** Yunxiang Zang, Alem W. Kahsai, Natalia Pakharukova, Li-yin Huang, Robert J. Lefkowitz

**Affiliations:** 1 Department of Medicine, Duke University Medical Center, Durham, North Carolina, USA; 2 Howard Hughes Medical Institute, Duke University Medical Center, Durham, North Carolina, USA; 3 Department of Biochemistry, Duke University Medical Center, Durham, North Carolina, USA

**Keywords:** C-Raf, arrestin, G protein–coupled receptors, allosteric activation, signal transduction, βarr, β-arrestin, CR, conserved region, CRD, cysteine-rich domain, ERK, extracellular signal–regulated kinase, GPCR, G protein–coupled receptor, GST, glutathione-*S*-transferase, MAPK, mitogen-activated protein kinase, MBP, myelin basic protein, MEK, Raf–MAPK extracellular signal–regulated kinase, MSP, membrane scaffold protein, M_2_V_2_R, M2 muscarinic receptor with phosphorylated tail of V2 vasopressin receptor, RBD, Ras-binding domain, SEC, size-exclusion chromatography, V_2_Rpp, V2 vasopressin receptor phosphopeptide

## Abstract

G protein–coupled receptors (GPCRs) convert external stimuli into cellular signals through heterotrimeric guanine nucleotide-binding proteins (G-proteins) and β-arrestins (βarrs). In a βarr-dependent signaling pathway, βarrs link GPCRs to various downstream signaling partners, such as the Raf–mitogen-activated protein kinase extracellular signal–regulated kinase–extracellular signal-regulated kinase cascade. Agonist-stimulated GPCR–βarr complexes have been shown to interact with C-Raf and are thought to initiate the mitogen-activated protein kinase pathway through simple tethering of these signaling partners. However, recent evidence shows that in addition to canonical scaffolding functions, βarrs can allosterically activate downstream targets, such as the nonreceptor tyrosine kinase Src. Here, we demonstrate the direct allosteric activation of C-Raf by GPCR–βarr1 complexes *in vitro*. Furthermore, we show that βarr1 in complex with a synthetic phosphopeptide mimicking the human V2 vasopressin receptor tail that binds and functionally activates βarrs also allosterically activates C-Raf. We reveal that the interaction between the phosphorylated GPCR C terminus and βarr1 is necessary and sufficient for C-Raf activation. Interestingly, the interaction between βarr1 and C-Raf was considerably reduced in the presence of excess activated H-Ras, a small GTPase known to activate C-Raf, suggesting that H-Ras and βarr1 bind to the same region on C-Raf. Furthermore, we found that βarr1 interacts with the Ras-binding domain of C-Raf. Taken together, these data suggest that in addition to canonical scaffolding functions, GPCR–βarr complexes directly allosterically activate C-Raf by binding to its amino terminus. This work provides novel insights into how βarrs regulate effector molecules to activate downstream signaling pathways.

G protein–coupled receptors (GPCRs), also called 7-transmembrane receptors, are the largest group of membrane proteins that regulate a multitude of physiological processes (1, 2). Approximately 35% of all Food and Drug Administration–approved drugs target GPCRs (3). In response to various external stimuli, such as light, hormones, and neurotransmitters, GPCRs initiate diverse cellular signaling processes through G protein–dependent and β-arrestin (βarr)-dependent pathways (4, 5). In the classical G protein–dependent pathway, agonist-bound GPCRs activate heterotrimeric guanine nucleotide-binding proteins (G proteins), leading to cellular response through a range of second messengers and other effectors (6, 7). The C-tail of GPCRs is phosphorylated by GPCR kinases (8). βarrs then bind to the phosphorylated receptor tail and engage with the receptor core, sterically block G protein coupling, and mediate the internalization of GPCRs (9, 10). Furthermore, βarrs coupling to the activated GPCR initiates alternative signaling pathways independent of or in concert with G proteins (5, 11). βarrs thus serve as adaptors and scaffold proteins that link GPCR to numerous signaling molecules, such as components of mitogen-activated protein kinase (MAPK) cascades and nonreceptor tyrosine kinase Src, and regulate various cellular functions, such as chemotaxis and apoptosis (12, 13, 14, 15).

MAPK cascades play a critical role in transmitting environmental signals into cellular responses (16). A typical MAPK cascade consists of three types of kinases: an MAP kinase kinase kinase, an MAP kinase kinase, and an MAPK (17). In the typical Raf–MAPK extracellular signal–regulated kinase (MEK)–extracellular signal-regulated kinase (ERK) module, activated C-Raf (MAP kinase kinase kinase) phosphorylates downstream MEK1 (also known as MAP kinase kinase), which then phosphorylates the ERK (MAPK). ERK then phosphorylates numerous effector proteins and regulates diverse physiological processes, such as cell growth, proliferation, and differentiation (18). C-Raf is the most upstream kinase in the cascade, and its activation is crucial for MAPK signaling. C-Raf consists of three conserved regions (CRs) (Fig. 1*A*). CR1, on the amino terminus, contains a Ras-binding domain (RBD) and a cysteine-rich domain (CRD); CR2 corresponds to a region rich in serine/threonine residues, whereas CR3 includes the catalytic domain on the carboxyl terminus (19). The activation of C-Raf is a complex process that involves Ras binding, regulatory phosphorylation, and protein–lipid interactions (19, 20).

**Figure 1:**
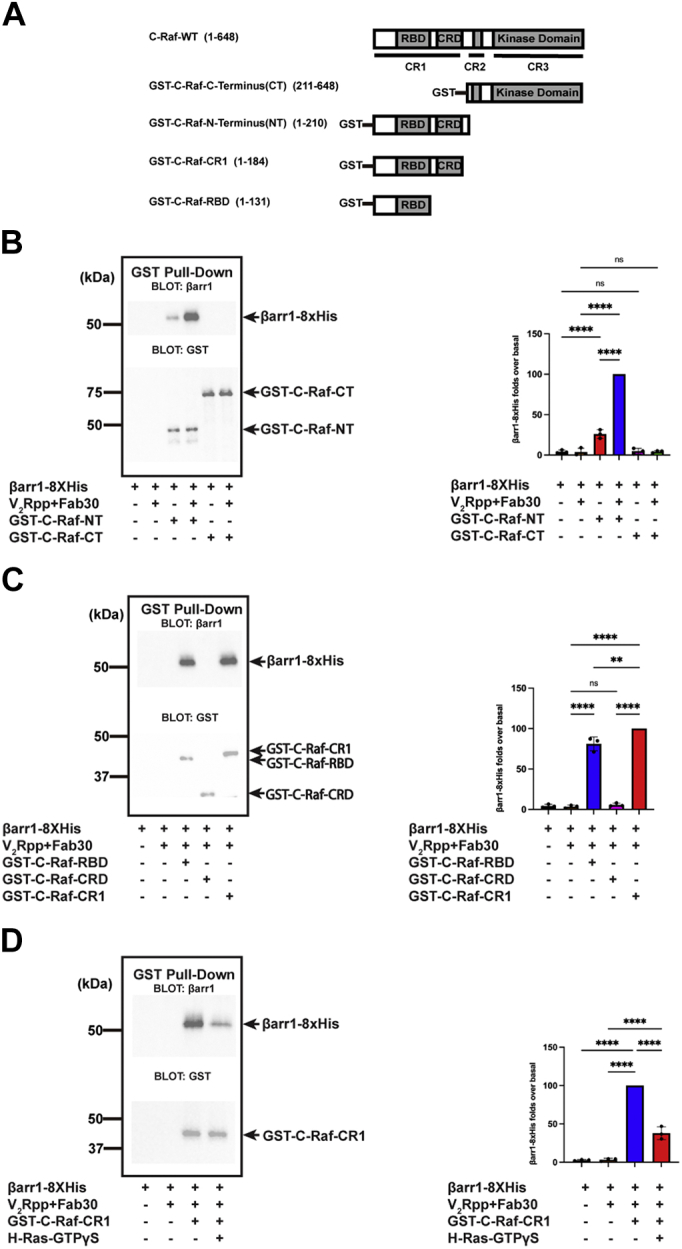
**β-arrestin 1 interacts with the amino terminus of C-Raf.***A*, schematic diagram of WT and different fragments of glutathione-*S*-transferase (GST)-tagged C-Raf. *B*, GST pull down of βarr1-8× His with GST-C-Raf amino terminus or GST-C-Raf carboxyl terminus. Representative Western blot using anti-βarr1 antibody (*upper panel*) or anti-GST antibody (*lower panel*) and quantification of βarr1-8× His binding. Data represent mean ± SD of three independent experiments. One-way ANOVA was performed to determine statistical differences (ns; ∗∗∗∗*p* < 0.0001). *C*, GST pull down of βarr1-8× His with GST-C-Raf-RBD, GST-C-Raf-CRD, or GST-C-Raf-CR1. Representative Western blot using anti-βarr1 antibody (*upper panel*) or anti-GST antibody (*lower panel*) and quantification of βarr1-8× His binding. Data represent mean ± SD of three independent experiments. One-way ANOVA was performed to determine statistical differences (ns; ∗∗*p* < 0.01; ∗∗∗∗*p* < 0.0001). *D*, GST pull down of βarr1-8× His (10 μM) with GST-C-Raf-CR1 in the presence of GTPγS-bound H-Ras (50 μM). Representative Western blot using an anti-GST antibody (*upper panel*) or anti-βarr1 antibody (*lower panel*) and quantification of βarr1-8× His binding. Data represent mean ± SD of three independent experiments. One-way ANOVA was performed to determine statistical differences (∗∗∗∗*p* < 0.0001). CR1, conserved region 1; CRD, cysteine-rich domain; ns, not significant; RBD, Ras-binding domain.

GPCR–βarr complexes interact with C-Raf to initiate MAPK cascade signaling (13, 14). Both the N and C domains of βarrs have been known to interact with C-Raf (21). However, the βarr-binding site in C-Raf has not been determined. βarrs have been demonstrated to act like typical scaffold proteins that bring together the components of MAPK cascade to facilitate signal transduction (13, 14). Surprisingly, recent studies showed that in addition to scaffolding functions, GPCR–βarr complexes may serve as allosteric modulators for downstream signaling partners such as kinase Src (22, 23). However, it is unclear whether βarrs can also allosterically activate C-Raf. Here, we show that βarr1, activated *via* either phosphorylated GPCR or the GPCR surrogate V_2_Rpp (a vasopressin receptor 2 phosphorylated C-terminal peptide with eight phosphates), binds C-Raf and allosterically activates it. Therefore, GPCR–βarr complexes not only function just as typical scaffold proteins for the C-Raf/MEK/ERK module but also play an active role in regulating MAPK signaling.

## Results

### βarr1 interacts with the amino terminus of C-Raf

To identify the βarr1-binding elements within C-Raf, we performed glutathione-*S*-transferase (GST) pull-down assays using GST-C-Raf fusion proteins and βarr1 (Fig. 1*A*). βarr1 coeluted with the GST-C-Raf amino terminus, and no βarr1 was detected for GST-C-Raf carboxyl terminus (Fig. 1*B*). These results show that GST does not interact with βarr1 and, therefore, βarr1 and C-Raf amino terminus interact specifically. Both free and active βarr1 bind C-Raf amino terminus; however, the binding was significantly enhanced in the presence of V_2_Rpp and the stabilizing antibody Fab30. The amino terminus of C-Raf encompasses both the RBD and CRD. To investigate the roles of both domains in the C-Raf–βarr1 interaction, GST-C-Raf-RBD, GST-C-Raf-CRD, and GST-C-Raf-CR1 (which contains the RBD and CRD) were used as bait proteins to perform the pull down with βarr1. Whereas GST-C-Raf-CRD pulled down no βarr1, GST-C-Raf-CR1 binds more βarr1 than GST-C-Raf-RBD (Fig. 1*C*). These results indicate that the RBD domain of C-Raf directly interacts with βarr1. In contrast, the presence of the CRD domain might be essential for optimal folding and stability of the RBD domain, thus contributing to the enhanced binding of βarr1. Interestingly, H-Ras, a small GTPase known to activate C-Raf, also interacts with the same domains of C-Raf (24). To investigate whether binding sites for H-Ras and βarr1 have any overlap, we set up an experiment where H-Ras loaded with GTPγS (a nonhydrolyzable analog of GTP) can compete with βarr1 to bind GST-C-Raf-CR1. With an excess of activated H-Ras, the interaction between GST-C-Raf-CR1 and βarr1 was significantly reduced (Fig. 1*D*), consistent with competitive binding of both βarr1 and H-Ras to the same region of C-Raf. Therefore, these data suggest that βarr1 binds to the amino terminus of C-Raf similarly as H-Ras.

### GPCR–βarr1 complexes allosterically activate C-Raf by interacting with its amino terminus

**Figure 2:**
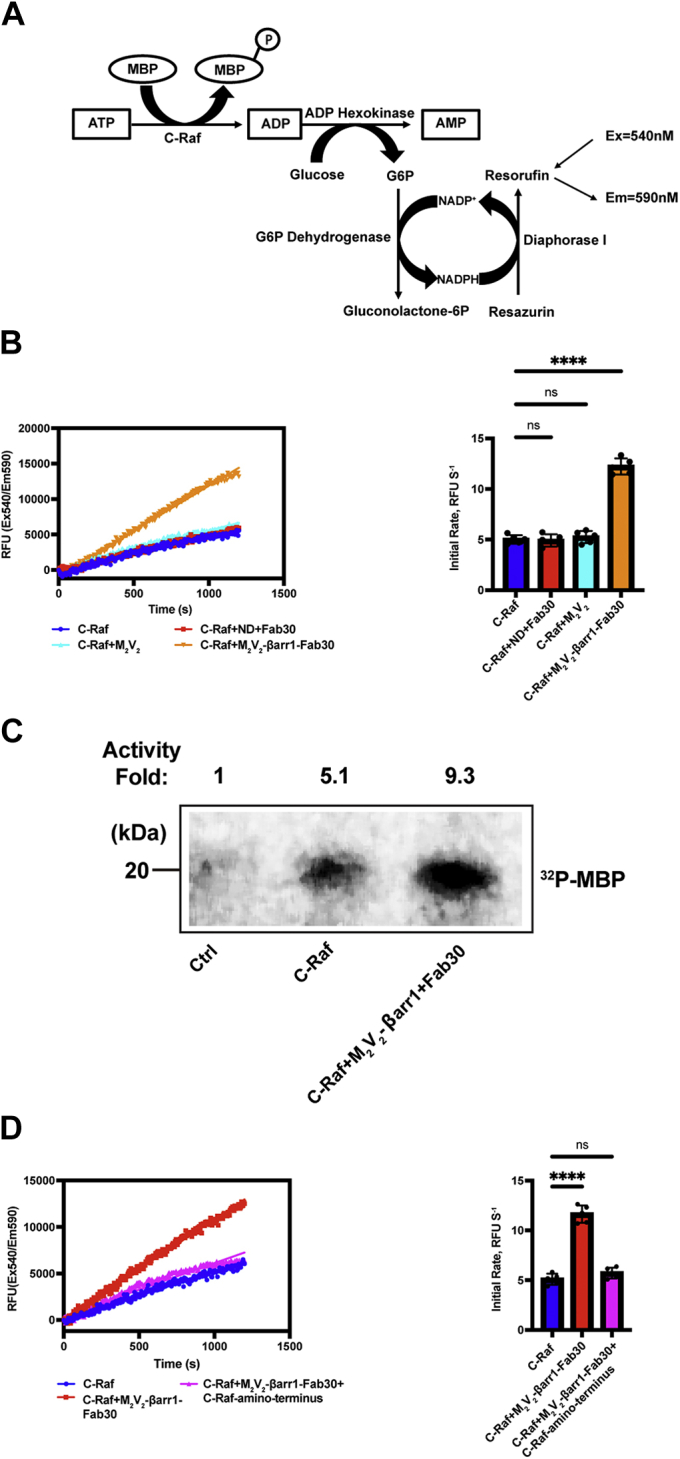
**GPCR–βarr1 complexes allosterically activate C-Raf by interacting with its amino terminus.***A*, schematic representation of enzyme-coupled fluorescence assay for quantification of C-Raf catalytic activity toward myelin basic protein (MBP). *B*, M_2_V_2_R–βarr1 complex allosterically activates C-Raf. *Left panel*, representative time courses of C-Raf activity in the presence of M_2_V_2_R–βarr1 complex. *Right panel*, calculated initial C-Raf reaction rate. As controls, empty MSP1D1E3 nanodisc (ND) and Fab30 or M_2_V_2_R alone were tested. Data represent mean ± SD of five independent experiments. One-way ANOVA was performed to determine statistical differences (ns; ∗∗∗∗*p* < 0.0001). *C*, C-Raf activity toward MBP was measured alone or in the presence of the M_2_V_2_R–βarr1 complex. MBP with the M_2_V_2_R–βarr1 complex alone serves as a control. The C-Raf was incubated with MBP and [γ-^32^P] ATP, and the radiolabel incorporated into MBP was analyzed by SDS-PAGE and autoradiography. A representative image of two independent experiments with similar results is shown. *D*, excess of C-Raf amino terminus (2.5 μM) blocks the C-Raf activation promoted by the M_2_V_2_R–βarr1 complex (500 nM). *Left panel*, representative time courses of C-Raf activity in the presence of excess C-Raf amino terminus. *Right panel*, calculated initial C-Raf reaction rate. Data represent as mean ± SD of five independent experiments. One-way ANOVA was performed to determine statistical differences (ns; ∗∗∗∗*p* < 0.0001). βarr1, β-arrestin 1; GPCR, G protein–coupled receptor; M_2_V_2_R, M2 muscarinic receptor with phosphorylated tail of V2 vasopressin receptor; ns, not significant.

GPCR–βarr complexes have been demonstrated to allosterically activate signaling partners such as the tyrosine kinase Src (22, 23). To investigate whether GPCR–βarr complexes allosterically regulate C-Raf activation, we used an enzyme-coupled fluorescence assay to measure the C-Raf activity in real time (Fig. 2*A*). To exclude the scaffolding function of βarr1, we measured the phosphorylation of myelin basic protein (MBP) instead of the physiological substrate MEK, by C-Raf. We used a chimeric M2 muscarinic receptor with a phosphorylated C-tail of the vasopressin 2 receptor (M_2_V_2_R) that confers tight binding to βarr1. M_2_V_2_R was reconstituted in ∼12-nm membrane scaffold protein (MSP) MSPD1E3 nanodiscs as this environment closely mimics a native membrane, and therefore, it represents a fully engaged M_2_V_2_R–βarr1 complex based on the previous cryo-EM study (25). GPCR–βarr complexes such as the one used here, M_2_V_2_R–βarr1 complex, are further stabilized by a synthetic antibody fragment Fab30. The C-Raf catalytic activity toward MBP significantly increased in the presence of the M_2_V_2_R–βarr1 complex, as shown in real time (Fig. 2*B*, *left*) and as calculated initial rates (Fig. 2*B*, *right*) of C-Raf activity. The empty nanodisc and Fab30 or M_2_V_2_R alone failed to increase the C-Raf activity. To further ascertain whether the M_2_V_2_R–βarr1 complex activates C-Raf, we utilized a complementary *in vitro* kinase assay using γ-^32^P labeled ATP to assess the phosphorylation of MBP by C-Raf in the absence or the presence of M_2_V_2_R–βarr1. We found that the M_2_V_2_R–βarr1 complex could increase the C-Raf activity *in vitro*, consistent with our enzyme-coupled fluorescence-based assay (Fig. 2*C*). Therefore, our findings reveal that the M_2_V_2_R–βarr1 complex allosterically activates kinase C-Raf. Next, we used an excess of the C-Raf amino terminus to test its ability to competitively inhibit the allosteric activation of C-Raf by M_2_V_2_R–βarr1. The allosteric activation of C-Raf is blocked by excess C-Raf amino terminus (Fig. 2*D*). These results reveal that the M_2_V_2_R–βarr1 complex allosterically activates C-Raf by interacting with its amino terminus. These data also demonstrate that βarr1 binds to the amino terminus of C-Raf.

### The interaction between GPCR phosphorylated C terminus and βarr1 is sufficient for the activation of C-Raf

**Figure 3:**
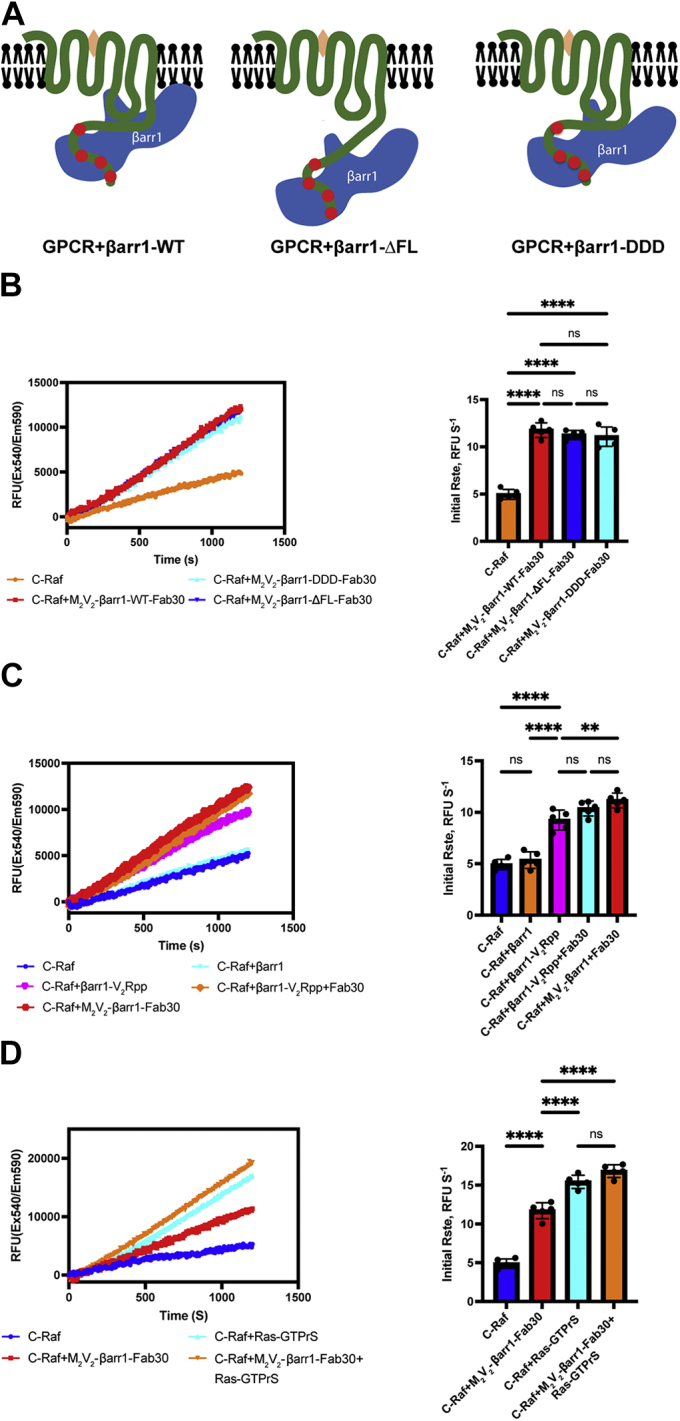
**The interaction between GPCR phosphorylated C terminus and βarr1 is sufficient to activate C-Raf.***A*, cartoon illustrating different conformations of GPCR–βarr1 complexes. *Left*, fully engaged GPCR–βarr1-WT complex (tail–core–lipid interactions); *middle*, GPCR–βarr1-ΔFL complex in “tail” conformation; *right*, GPCR–βarr1-DDD complex that lacks the interaction between βarr1 with the lipid bilayer (*khaki*, Ligand). *B*, C-Raf is allosterically activated by different conformations of M_2_V_2_R–βarr1 complexes. *Left panel*, representative time courses of C-Raf activity in the presence of M_2_V_2_R–βarr1-WT, M_2_V_2_R–βarr1-ΔFL, or M_2_V_2_R**–**βarr1-DDD complexes. *Right panel*, calculated initial C-Raf reaction rate. Data represent mean ± SD of five independent experiments. One-way ANOVA was performed to determine statistical differences (ns; ∗∗∗∗*p* < 0.0001). *C*, βarr1–V2Rpp–Fab30 complex allosterically activates C-Raf. *Left panel*, representative time courses of C-Raf activity in the presence of free βarr1, βarr1**–**V_2_Rpp, βarr1**–**V_2_Rpp**–**Fab30, or M_2_V_2_R–βarr1 complexes. *Right panel*, calculated initial C-Raf reaction rate. Data represent mean ± SD of five independent experiments. One-way ANOVA was performed to determine statistical differences (ns; ∗∗*p* < 0.01; ∗∗∗∗*p* < 0.0001). *D*, C-Raf is allosterically activated by the M_2_V_2_R**–**βarr1 complex and activated H-Ras. *Left panel*, representative time courses of C-Raf activity in the presence of the M_2_V_2_R**–**βarr1 complex (500 nM), H-Ras-GTPγS (500 nM), or both M_2_V_2_R**–**βarr1 and H-Ras-GTPγS (500 nM each). *Right panel*, calculated initial C-Raf reaction rate. Data represent mean ± SD of five independent experiments. One-way ANOVA was performed to determine statistical differences (ns; ∗∗∗∗*p* < 0.0001). βarr1, β-arrestin 1; GPCR, G protein–coupled receptor; M_2_V_2_R, M2 muscarinic receptor with phosphorylated tail of V2 vasopressin receptor; ns, not significant; V2Rpp, V2 vasopressin receptor phosphopeptide.

Previous studies revealed that GPCR–βarr complexes adopt two different conformations: in the “core” conformation, βarrs bind to the phosphorylated C terminus and the transmembrane bundle of GPCR; in the “tail” conformation, βarrs bind only to the phosphorylated GPCR C terminus (26). Recent structural studies show that the C-edge of βarr1 interacts with the lipid bilayer and functions as a membrane anchor (Fig. 3*A*) (25, 27). By removing the βarr1 “finger loop,” which is required for interactions with the receptor core, we obtained a M_2_V_2_R–βarr1-ΔFL complex exclusively in the “tail” conformation (28). By introducing mutations (L335D/L338D/S340D) in the C-edge of βarr1, we formed a M_2_V_2_R–βarr1-DDD complex that lacks interactions with the lipid bilayer (Fig. 3*A*). Both M_2_V_2_R–βarr1-ΔFL and M_2_V_2_R**–**βarr1-DDD complexes showed similar ability to enhance C-Raf activity compared with the M_2_V_2_R**–**βarr1-WT complex (Fig. 3*B*). These results suggest that the interactions neither between the GPCR transmembrane core and βarr1 nor between the C-edge of βarr1 and the lipid bilayer are necessary for the allosteric activation of C-Raf by βarr1. These findings suggest that the binding of the phosphorylated GPCR C terminus to βarr1 plays a critical role in the allosteric activation of C-Raf. To test this, V_2_Rpp was used to activate βarr1 *in vitro*. We found that the V_2_Rpp-bound active βarr1 could allosterically activate C-Raf, whereas inactive βarr1 failed to do so. These results also show that V_2_Rpp alone was sufficient to activate C-Raf, whereas Fab30 alone did not have a significant effect to this end (Fig. 3*C*). These results demonstrate that the interaction between the phosphorylated GPCR C terminus and βarr1 is necessary and sufficient for the allosteric activation of C-Raf.

### The extent of allosteric activation of C-Raf by GPCR–βarr complexes is comparable to that by activated H-Ras

Active GTP-bound H-Ras plays a critical role in the activation process of C-Raf (19, 20). To compare the allosteric activation ability between GPCR–βarr complexes and activated H-Ras on C-Raf, we measured the C-Raf enzymatic activity in the presence of the M_2_V_2_R–βarr1 complex or GTPγS-bound H-Ras. The kinetics and calculated initial rate of C-Raf activity show that both M_2_V_2_R–βarr1 and GTPγS-bound H-Ras enhance the catalytic activity of C-Raf toward MBP (Fig. 3*D*). The activation effect of GTPγS-bound H-Ras is slightly but significantly higher than that of the M2V2R–βarr1 complex. C-Raf activity is enhanced but not significantly when both the M2V2R–βarr1 and GTPγS-bound H-Ras are added together (Fig. 3*D*). In our study, the nonhydrolyzable ATP analog ATPγS was used during the purification process to stabilize C-Raf. These results show that the M_2_V_2_R–βarr1 complex or GTPγS-bound H-Ras allosterically activate ATPγS-bound C-Raf in a comparable extent. Taken together, our findings demonstrate that in addition to the canonical scaffolding function, the GPCR–βarr complexes serve as direct allosteric activators of C-Raf.

## Discussion

βarrs have been demonstrated to interact with the C-Raf/MEK/ERK cascade after coupling to multiple GPCRs and thereby mediate activation of this pathway either independent of or codependent on G proteins (11, 15). Previous findings have suggested that C-Raf interacts with both the N and C domains of βarr1 (21, 29, 30). A model of GPCR/βarr/ERK module complexes predicted by protein–protein dockings suggested that βarr1 binds to the RBD of C-Raf (31). In the current study, using GST pull-down assays, we demonstrated that the RBD domain of C-Raf interacts with βarr1. In addition, we found that GTPγS-bound activated H-Ras competes with βarr1 for binding to C-Raf. Since activated H-Ras is thought to bring C-Raf to the plasma membrane (32), perhaps GPCR–βarr complexes might play a similar role as an alternate means of conveying the enzyme to the cell membrane during the activation process of C-Raf.

Previously, βarrs have been considered adaptors and scaffold proteins that link the receptors to different signaling partners and bring various signaling components into close contact to facilitate the signal transduction process (15). However, recent studies have shown that βarr1 can play a more active role in signaling by allosterically activating its binding partner, Src kinase (22, 23). However, for the GPCR–βarr-dependent MAPK signaling pathway, the mechanistic details of how C-Raf is regulated to initiate the signaling cascade remained unclear. Previous findings indicate that βarr-dependent MAPK signaling might be a Ras-independent process (14). In the present study, we found that the active form of βarr1 allosterically activates C-Raf. Both the “tail” and “core” conformations of the M_2_V_2_R–βarr1 complex enhance the enzymatic activity of C-Raf. Our results indicate that the interaction between βarr1 and the phosphorylated GPCR C terminus is sufficient to activate C-Raf. The precise mechanism by which a GPCR–βarr complex activates C-Raf must be the subject of future studies.

Scaffold proteins facilitate signal transduction through tethering signaling components into specific complexes. Recent studies reveal that several scaffold proteins could allosterically regulate their partner molecules in addition to tethering (22, 23, 33). In the current study, we found that in addition to the canonical scaffolding, GPCR–βarr complexes also allosterically activate C-Raf. GPCR–βarr complexes perform the canonical scaffolding and allosteric regulation roles by interacting with the amino terminus of C-Raf. The canonical scaffolding and allosteric regulation processes are interdependent and synergistic. Through both tethering and allosteric mechanisms, GPCR–βarr complexes might enhance the efficiency and specificity of βarr-dependent MAPK signaling. Furthermore, GPCR–βarr complexes might initiate the MAPK signaling through a Ras-independent manner and activate a prolonged MAPK response. It is likely that GPCR–βarr complexes may allosterically activate other components in the MAPK cascade. Overall, our results indicate that the active form of βarr1 interacts with the amino-terminal regulatory region of C-Raf and allosterically enhances the basic catalytic activity of C-Raf.

## Experimental procedures

### Constructs

The construct expressing FLAG-M2R with C-terminal sortase ligation consensus sequence (LPETGGH) followed by a 6× His-tag has been previously described (34). WT Human C-Raf (residues 1–648) and C-Raf-carboxyl terminus (residues 211–648) were cloned into pVL1392 with an N-terminal GST tag. Human C-Raf-amino terminus (residues 1–210), C-Raf-CR1 (residues 1–184), C-Raf-RBD (residues 1–131), and C-terminal 8× His-tagged rat βarr1 (βarr1-8× His) were cloned into pGEX-4T1. The plasmids encoding βarr1-ΔFL, βarr1-DDD, and Fab30 were reported previously (25, 35). Plasmid expressing mouse H-Ras was a generous gift from Julian Downward (Addgene; plasmid 55653).

### Protein expression and purification

WT human C-Raf and C-Raf-carboxyl terminus were expressed and purified as previously described (36). Briefly, full-length human C-Raf or C-Raf-carboxyl terminus with an N-terminal GST tag were expressed in sf9 cells. The proteins were extracted and purified on glutathione sepharose beads (GoldBio). Then the GST tag was cleaved off by thrombin protease. Finally, C-Raf was further purified by size-exclusion chromatography (SEC). GST-C-Raf-amino terminus, GST-C-Raf-CR1, GST-C-Raf-RBD, and GST-C-Raf-CRD were expressed in *Escherichia coli* BL21 (DE3) cells and purified on glutathione sepharose beads (GoldBio) followed by further purification using SEC. Expression and purification of Fab30 (37), H-Ras (38), and WT βarr1 and its variants (39) have been described previously. Expression and purification of FLAG-M2R with C-terminal sortase ligation consensus sequence (LPETGGH) followed by a 6× His-tag was performed as previously described (34).

### Sortase ligation and high-density lipoprotein reconstitution

The synthetic phosphopeptide GGG-V_2_Rpp (GGGARGRpTPPpSLGPQDEpSCpTpTApSpSpSLAKDTSS) was ligated on the C terminus of M2R by enzyme sortase as previously described (34). The high-density lipoprotein reconstitution of M_2_V_2_R was performed as previously described (25). In short, M_2_V_2_R (5 mM) was incubated with twofold molar excess atropine at 4 °C for 30 min. Subsequently, M_2_V_2_R (5 mM) was mixed with a 3:2 M ratio of 8 mM 1-palmitoyl-2-oleoyl-glycero-3-phosphocholine with 1-palmitoyl-2-oleoyl-*sn*-glycero-3-phospho-(1′-rac-glycerol) and 80 μM MSP1D1E3 on ice for 1 h. Bio-Beads (Bio-Rad) were added to remove the detergent, and the mixture was incubated overnight at 4 °C. High-density lipoprotein-M_2_V_2_R was further purified by M1-FLAG and SEC.

### Enzyme-coupled fluorescence C-Raf kinase assay

C-Raf activity regulated by M_2_V_2_R–βarr1 complexes or activated H-Ras was measured with a Kinase/ADP kit (Fujifilm Wako Chemicals). In short, 100 nM C-Raf was mixed with 5 μM MBP (MilliporeSigma) as a substrate in the reaction buffer (20 mM Hepes, pH 7.4, 150 mM NaCl, 5 mM MgCl_2_, 0.5 mM Tris(2-carboxyethyl)phosphine hydrochloride). ATP with the final concentration of 20 μM was added to initiate the reaction. Same volume of commercially supplied “Detection solution” was added to the reaction, then the fluorescence intensity (excitation of 540/emission of 590) was measured continuously. The concentration of βarr1 or M_2_V_2_–βarr1 complexes in all experiments was 500 nM.

### GST pull-down assay

About 10 μM βarr1-8× His was incubated with 30 μM V_2_Rpp and 30 μM Fab30 at room temperature for 30 min. About 7 μM GST-C-Raf-RBD or other GST-tagged C-Raf variants were then added to the reaction mixture and incubated for 1 h at room temperature. Subsequently, 10 μl of Glutathione Sepharose beads (GoldBio) were added and incubated for another hour at 4 °C with end-to-end rotation. The beads were collected and washed three times using wash buffer (20 mM Hepes, pH 7.4, 150 mM NaCl). Finally, the proteins were eluted in elution buffer (20 mM Hepes, pH 8.0, 150 mM NaCl, and 20 mM reduced glutathione) and visualized by Western blotting using anti-GST antibody (Cytiva; RPN1236) and anti-βarr1 antibody (Cell Signaling; 30036).

### *In vitro* [γ-^32^P]ATP C-Raf kinase assay

The kinase activity of C-Raf was tested with MBP (MiliporeSigma). To measure C-Raf kinase activity *in vitro*, purified C-Raf protein (100 nM) was incubated with [γ-^32^P] ATP (20 μM, 8000 cpm/pmol) and MBP (5 μM) at 30 °C for 30 min. Incorporation of ^32^P into MBP was analyzed by SDS-PAGE and autoradiography.

## Data availability

All data presented are available upon request from Robert J. Lefkowitz (lefko001@receptor-biol.duke.edu).

## Conflict of interest

The authors declare that they have no conflicts of interest with the contents of this article.
